# Elegant Display of Chemicals

**Published:** 1878-11-20

**Authors:** 


### ELEGANT DISPLAY OF CHEMICALS.

During the late Fair Exhibition held in this city, our attention was called to the finest display of chemicals it was ever our pleasure to witness. They were from the celebrated Laboratory of Powers & Weightman, of Philadelphia, Pa., and were placed on exhibition by the enterprising drug-firm of Pemberton, Samuels & Reynolds, of Atlanta, Ga.

It remains for us to say that we can heartily commend Pemberton, Samuels & Reynolds to our friends as honest conscientious druggists and business men, who will always give them the purest and best medicines to be obtained. We know them personally and give testimony accordingly. W. T. G.

